# Molecular docking, a tool to determine interaction of CuO and TiO_**2**_ nanoparticles with human serum albumin

**DOI:** 10.1016/j.bbrep.2016.03.004

**Published:** 2016-03-11

**Authors:** Sandesh Chibber, Irshad Ahmad

**Affiliations:** a Institute of Life Sciences, School of Science and Technology, Ahmedabad University, Ahmedabad 380009, India; b Department of Biochemistry, F/O Life Sciences, Aligarh Muslim University, Aligarh 202002, India

**Keywords:** CuO NP, TiO_2_ NP, HSA, Subdomain III A, Suldow site I, Suldow site II

## Abstract

**Background:**

We study the human serum albumin (HSA) protein-CuO nanoparticle interaction to identify the specific binding site of protein with CuO nanoparticles by molecular docking and compared it with HSA-TiO_2_ nanoparticle interaction.

**Methods:**

The protein structural data that was obtained using Autodock 4.2.

**Results:**

In case of CuO np-HSA interaction, the distances from the centre of Subdomain IIIA to Arg-472 is 2.113 Å and Lys 475, Glu 492, Ala 490, Cys 487, Ala 490 are the bound neighbouring residues with Lys 475, Glu 492 at aliphatic region. The binding energy generated was −1.64 kcal mol^−1^. However, for TiO_2_ nanoparticle, the binding region is surrounded by Arg 257, Ala 258, Ser 287, His 288, Leu 283, Ala 254, Tyr 150 (subdomain II A) as neighbouring residue. Moreover, Glu 285, Lys 286 forms aliphatic grove for TiO_2_-HSA, Ser-287 at the centre region form hydrogen bond with nanoparticle and Leu 283, Leu 284 forming hydrophopobic grove for TiO_2_ nanoparticle-HSA interaction. The binding energy generated was −2.47 kcal mol^−1^.

**Conclusions:**

Analysis suggests that CuO bind to suldow site II i.e subdomain III A of HSA protein where as TiO_2_ nanoparticle bind to suldow site I i.e subdomain IIA of HSA protein.

**General significance:**

The structural information that derives from this study for CuO and TiO_2_ nanoparticles may be useful in terms of both high and low-affinity binding sites when designing these nanoparticles based drugs delivery system.

## Highlights

• Analysis suggests that CuO bind to suldow site II (subdomain III A) of HSA protein.
• TiO_2_ nanoparticle binds to suldow site I (subdomain II A) of HSA protein.
• The structural information is helpful in order to avoid binding to HSA.
• The structural information makes use of CuO and TiO_2_ NPs depot function for the delivery purpose.

## 1 Introduction

Human serum albumin (HSA) is one of the important transport proteins in the human blood and can bind to any ligand that injected into blood stream thus play important role in drug delivery system. Structurally, a single polypeptide chain of HSA consists of 585 amino acids. HSA consists of three homologous domains (I, II, and III) and each comprised of subdomain A and B. The subdomains IIA and IIIA of HSA consists of high affinity ligand binding sites and is referred as Sudlow's site I and II, respectively [1], [2].

The high surface areas and unusual crystal morphologies endue CuO NPs with antimicrobial activity. CuO NPs are most frequently used as antimicrobial (antiviral, antibacterial, antifouling, antifungal), antibiotic treatment alternatives, nanocomposite coating, catalyst, lubricants. Also, used in applications like gas sensors, solar energy conversion, electrode material in lithium ion batteries, as field emitter, and as a heterogeneous catalyst [3]. TiO_2_ NPs drive a strong interest, subsequently intensive experimental and theoretical studies, owing to its unique photocatalytic properties, excellent biocompatibility, and high chemical stability. TiO_2_ NPs are used widely in biomedical applications that include the photodynamic therapy for cancer treatment, drug delivery systems, cell imaging, biosensors for biological assay, and genetic engineering. High physical and chemical stability of CuO and TiO_2_ nanoparticles renders their extreme use in catalytic applications [4]. However, Nanotoxicology is come forth in the field of toxicology to address the gaps in knowledge and adverse health effects associated with CuO and TiO_2_ nanomaterials. CuO NPs exposure results in significantly elevated level of antioxidant enzymes. CuO NPs has also been found to induce hepatotoxicity and nephrotoxicity. CuO NPs can equally exhibit neurotoxicity and genotoxicity [5], [6], [7], [8]. TiO_2_ NPs has also been found to induced similar toxicity but to lesser extent as compared to CuO NPs [9], [10], [11], [12]. Thus, it is very important to understood interaction mechanism of CuO NPs and TiO_2_ NPs with serum albumin and accordingly reduces CuO NPs and TiO_2_ NPs vulnerability in the system thereby minimizing its toxic effects.

The binding affinity of a ligand with serum albumin helps us to understand its bioavailability, distribution, and elimination from the body. HSA can bind a notable variety of drugs affecting their delivery and efficacy and ultimately altering the drug’s pharmacokinetic and pharmacodynamic properties. Additionally, HSA is widely used in clinical assumptions as a drug delivery system due to its potential for improving targeting while decreasing the side effects of drugs. Nanoparticles such as CuO and TiO_2_ are widely used in medical application such as drug delivery system [13], [14]. Subsequently, the affinity between HSA and nanoparticles as legend becomes crucial interest to study in order to determine nanoparticles binding potential with circulating protein. The binding study conducted through molecular docking can help to infer the duration of its half-life that consequently reveals the efficacy of nanomedication [1]. Drug delivery mechanism is propagated through serum albumin. In order to introduce CuO NPs and TiO_2_ NPs in nanodelivery it is most worthy for the researchers to be aware about interaction sites and the region involve in binding with serum albumin so that nanoparticles itself may not become inhibitor for the drug binding sites. Therefore, present study conducted on the comparison of binding affinity of serum albumin with CuO and TiO_2_ nps. Thus, it may provide valuable information concerning their therapeutic efficacies.

## 2 Methods

Molecular docking studies were carried out using AutoDock 4.2 tool to predict the preferred binding mode and binding sites of TiO_2_ and CuO with HSA. The structure of TiO_2_ and CuO was drawn using ACD/ChemSketch and its geometry was optimized by combine use of Gaussian 03 program and Autodock 4.2. The crystal structure of HSA (PDB ID: 1E7I) was obtained from Protein Data Bank [15]. Before docking analysis Hetatm were removed from the protein and the energy was minimized by SPDBV-Swiss-pdbviewer. For docking calculations, Lamarckian genetic algorithms (LGA) were used and grid parameters were set as 126×126×126, with a spacing of 1 Å, in AutoDock 4.2. To determine the preferred binding sites on HSA, TiO_2_ and CuO molecules were allowed to move within the whole region of HSA via 50 runs to obtain the possible binding gesture. The output from AutoDock was further analyzed with PyMOL and UCSF Chimera software package [16], [17].

## 3 Results

### 3.1 Different residues in HSA protein and their distances from tryptophan in case of CuO np-HSA interaction

**Fig. 1:**
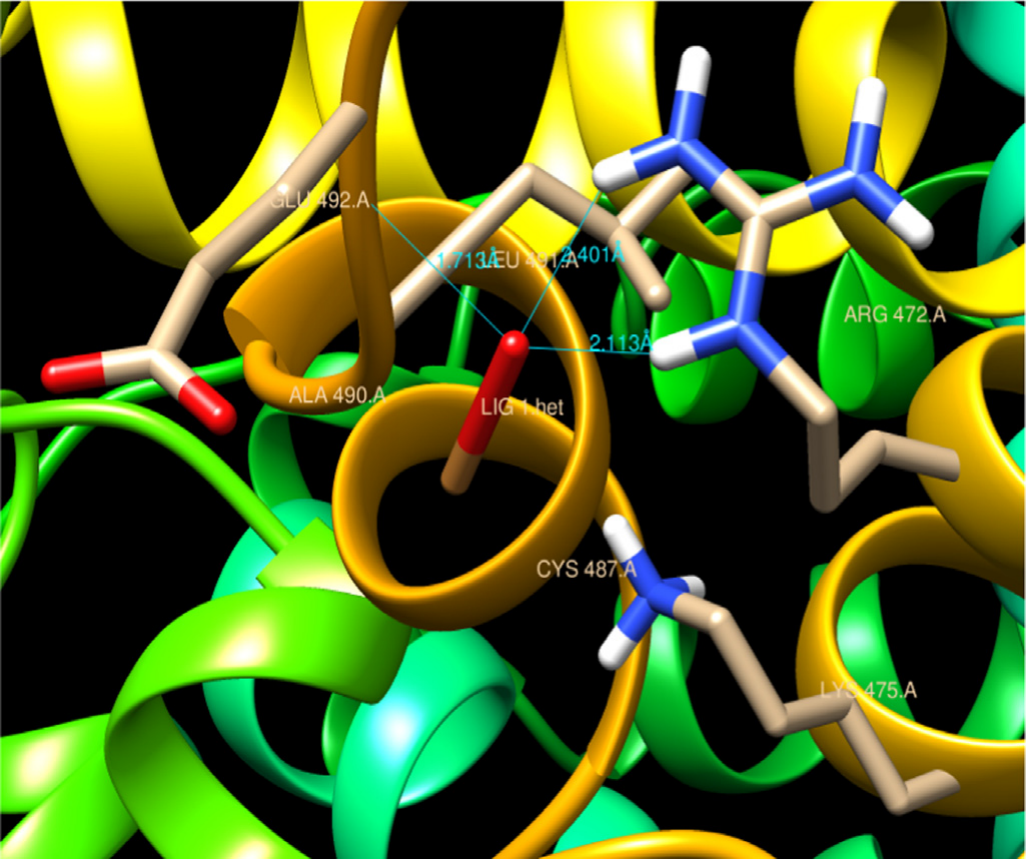
Residual interactions at the HSA-CuO nanoparticle interface in HSA. Schematic representations of different residues that are involve in binding with CuO NPs and their distances obtained from central subdomain III A.

The preferable binding of CuO nanoparticles to HSA protein obtained was through polar residue Arg 472 residue and through Lys 475, Glu 492, Ala 490, Cys 487, Ala 490 bond formation. The measured distances between the surfaces of the CuO np and Arg residue (subdomain IIIA) site II obtained were 2.113 Å, 2.410 Å, with Glu having 1.713 Å as shown in Fig. 1. Therefore, these sites would be the probable binding sites of HSA with CuO nanoparticles.

### 3.2 Geometrical accommodation and the chemical environment of domain in HSA in case of CuO np-HSA binding

**Fig. 2:**
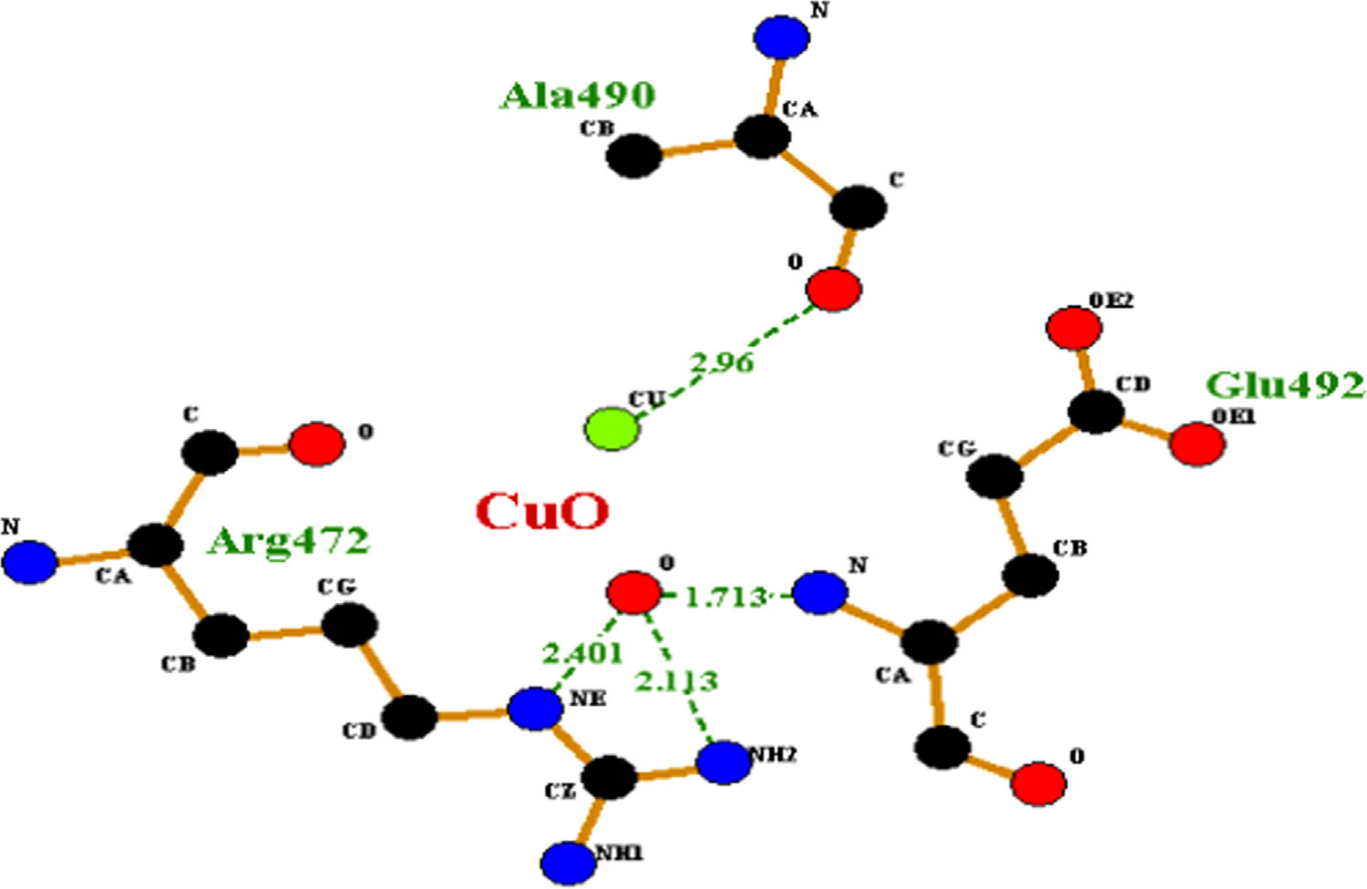
Graphical representation of HSA-CuO nanoparticle complex. Hydrophobic moieties involve in interactions with CuO NPs are shown as graphical representation.

**Fig. 3:**
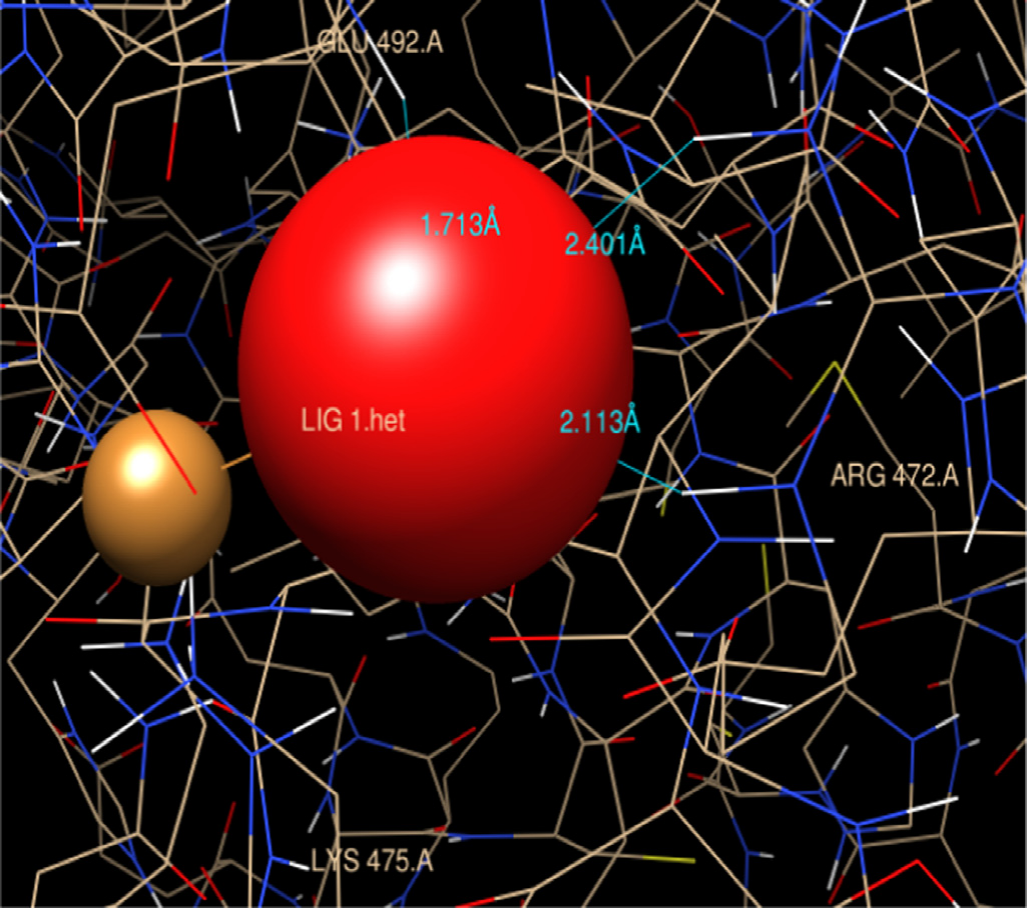
Aliphatic region identification of HSA-CuO nanoparticle interaction. The surrounding residue of the binding region of HSA that forms grove with CuO NPs.

4 hydrogen bonds were predicted involving hydrogen atoms from three different amino acid residues of HSA (Arg-472, Gln-492 and Ala 490) as showed in Fig. 2. Lys 475, Glu 492 residue are charged grove of HSA bind with CuO nanoparticle as depicted in Fig. 3.

### 3.3 Different residues in HSA protein and their distances from surface of TiO_2_ np-HSA interaction

**Fig. 4:**
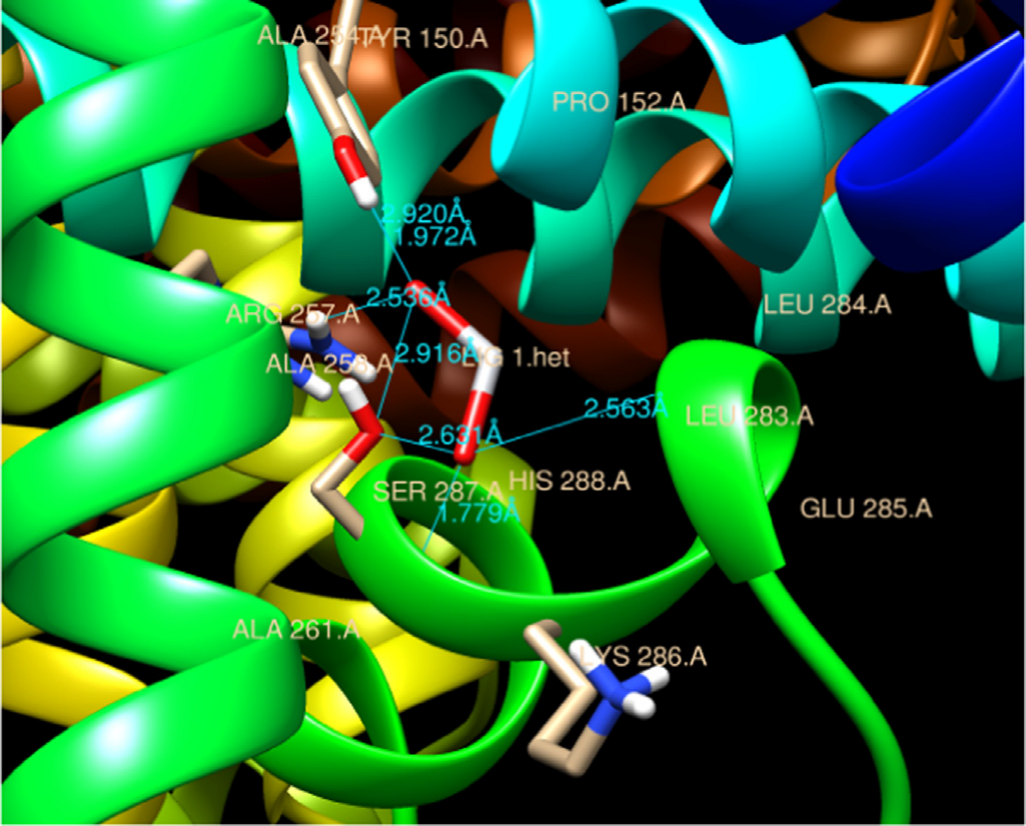
Residual interactions at the HSA-TiO_2_ nanoparticle interface in HSA. Schematic representations of different residues in HSA protein and their distances from centre subdomain II A.

The preferable binding of TiO_2_ nanoparticles to HSA protein obtained was through polar hydrophilic amino acids Arg 257 residue and thorugh Ala 258, Ser 287, His 288, Leu 283, Ala 254, Tyr 150 form bond formation with TiO_2_ np. The measured distances between the surfaces of the TiO_2_ np and Arg residue (subdomain II A) obtained were 2.536 Å. However, 2.916 Å, 2.631 Å, 1.779 Å, 2.563 Å, 2.920 Å and 1.972 Å is the bond length with respective amino acids of bound region as shown in Fig. 4. Therefore, these sites would be the probable binding sites of HSA with TiO_2_ nanoparticle.

### 3.4 Geometrical accommodation and the chemical environment of domain in HSA in case of TiO_2_ np-HSA binding

**Fig. 5:**
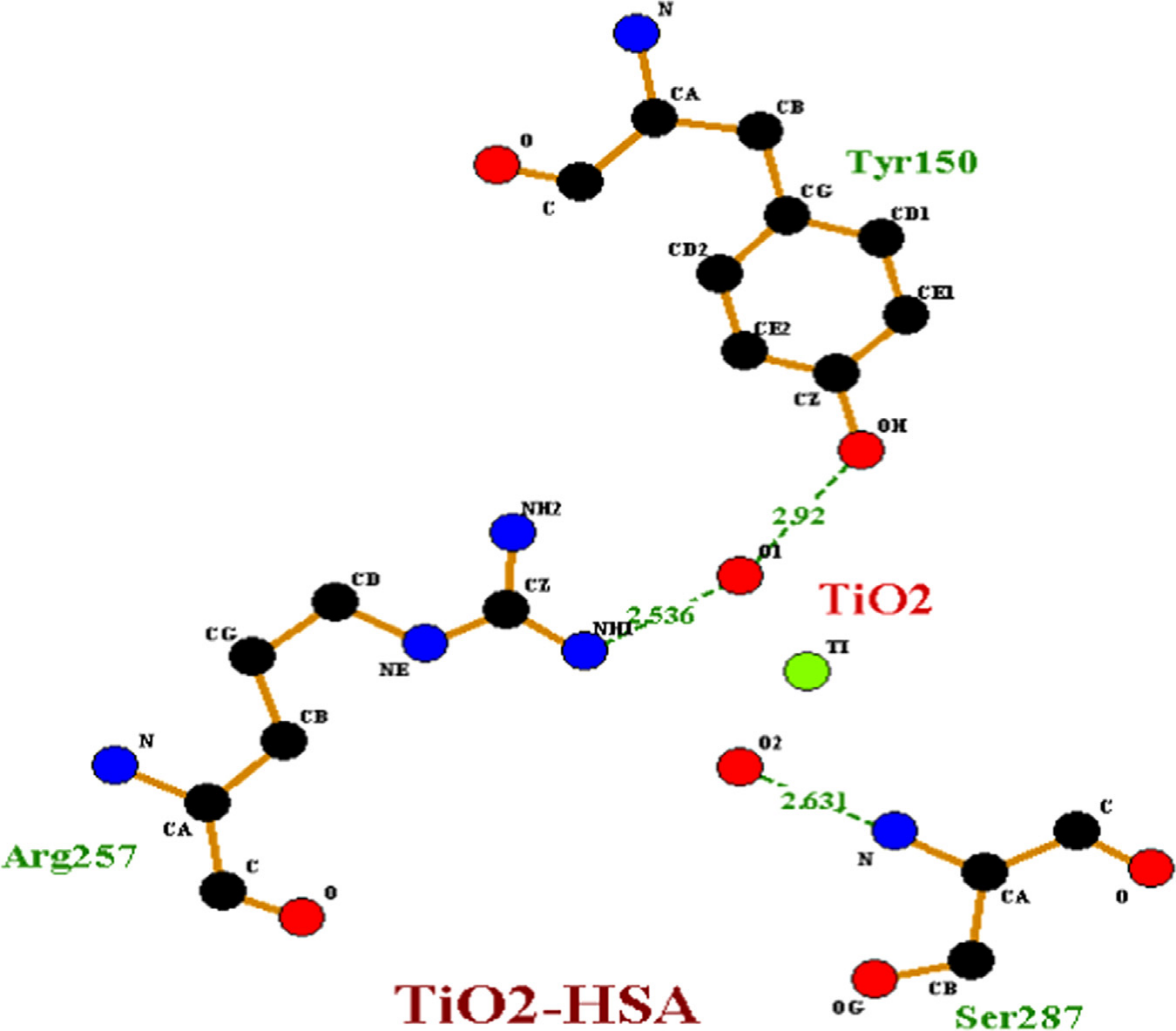
Graphical representation of HSA-TiO_2_ nanoparticle complex. Residues involved in the hydrophobic interactions are shown in graphical form.

**Fig. 6:**
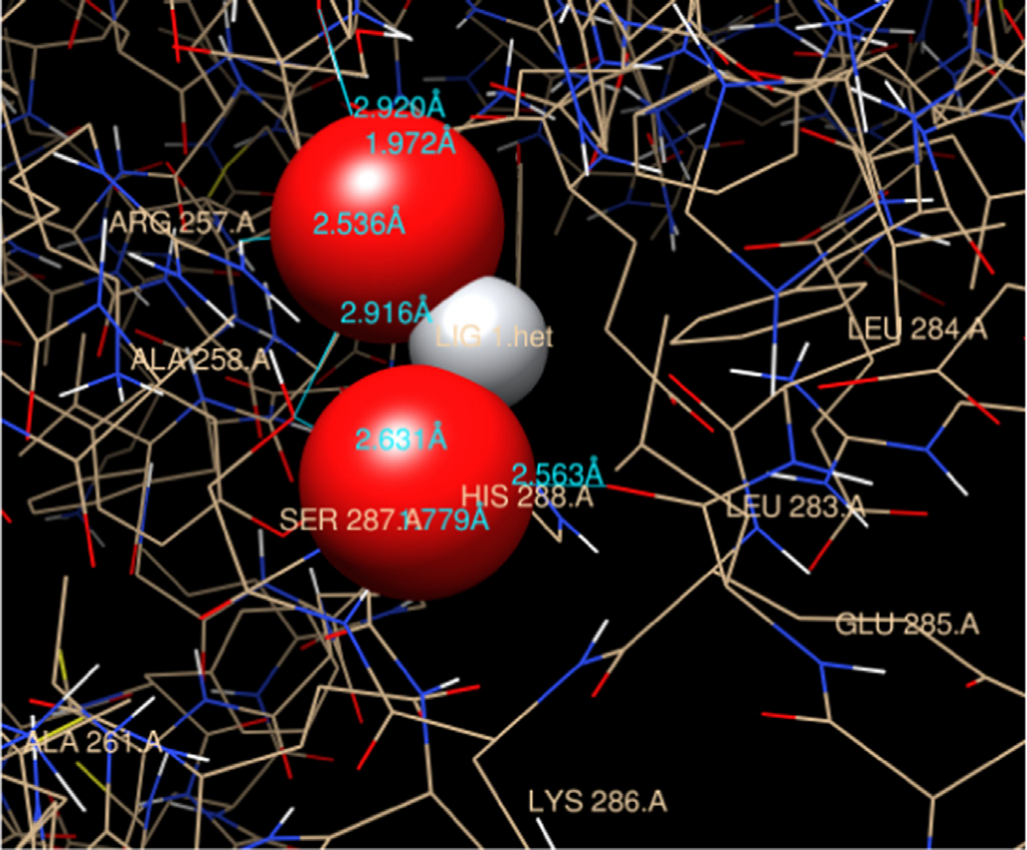
Aliphatic region of HSA-TiO_2_ nanoparticle complex. The surrounding residue of HSA that forms grove with TiO_2_ NPs.

4 hydrogen bonds were predicted involving hydrogen atoms from three different amino acid residues of HSA (Tyr 150, Ser-287 and Arg 257) as shown in Fig. 5. Leu 283, Leu 284, Glu 285, Lys 286 residue form hydrophobic pocket of HSA that bind with TiO_2_ nanoparticle as depicted in Fig. 6.

### 3.5 Binding energy predicted for CuO np-HSA interaction and TiO_2_ np-HSA interaction

The predicted binding models of CuO with the lowest docking energy (−1.64 kcal mol^−1^) at site II of HSA were used for binding orientation analysis. Whereas, in case of TiO_2_ nanoparticle the lowest docking energy obtained was −2.47 kcal mol^−1^ (Table 1) at the binding region.

**Table 1:** Binding energy obtained for CuO nanoparticle HSA interaction and TiO_2_ nanoparticle HSA Interaction by molecular docking approach. Binding energy generated is higher in case of CuO NPs as compared to TiO_2_ NPs HSA interaction.

	**TiO_2_**	**CuO**
Binding energy	−2.47 kcal mol^−1^	−1.64 kcal mol^−1^

## 4 Discussion

Amino acid sequence of human serum albumin (HSA) consists of 18 tyrosines, 6 methionines, 1 tryptophan (Trp 214), 17 disulfide bridges, and only one free thiol (Cys 34) residue. HSA comprises of three homologous domains (I, II, and III) that assemble to form a cordate molecule. The hydrophobic pockets at subdomains IIA and IIIA is the main regions of ligand interaction with IIIA having the highest affinity for the interaction [2], [18]. In our study the whole region of HSA was selected in order to predict binding of HSA with CuO and TiO_2_ nanoparticle for the autodock. Many ligands bind specifically to HSA, either in site I (located at subdomain IIA) or in site II (located at subdomain IIIA). In our case CuO bind to site II, subdomain III A and TiO_2_ nanoparticle bind to site I located at subdomain II A.

The single tryptophan of HSA at residue 214 has been used extensively for binding studies [19]. From the protein structural data the distances from the centre of subdomain IIIA to Arg-472 (subdomain III A) was found to be 2.113 Å whereas on entering the grid for TiO_2_ nanoparticle the region predicated by Autodock 4.2 is Arg 257 (subdomain IIA) with bond length of 2.536 Å. The structural information that derives from this study for CuO and TiO_2_ may be useful for designing these nanoparticles based drug delivery system considering in terms of both high and low-affinity binding sites. This is helpful in order to avoid binding to HSA, or to make use of its depot function for the delivery purpose.

The attachment of CuO nanoparticles at the site II may be explained by considering the geometrical arrangement (Arg-472, Gln-492 and Ala 490) surrounded by and the environment of charged pocket Lys 475, Glu 492 of the domain (subdomain III A) in HSA. However in case of TiO_2_ nanoparticle structural binding residue involved were Arg 257, Ala 258, Ser 287, His 288, Leu 283, Ala 254, Tyr 150 at the interior hydrophobic binding pocket and comprise loop helix features of HSA at subdomain IIA. The other residues which was docked were Ser-287, Leu 283, Leu 284, Glu 285, Lys 286 this show that TiO_2_ np interact with both the cluster of polar residues at suldow site II (subdomain II A) of HSA protein [13]. Upon CuO nanoparticle binding, Lys 475 from subdomain III A moves to interact with the Cu moiety of the nanoparticle bound to a site that straddles domains II. However, TiO_2_ nanoparticle docked with Lys 286 at subdomain II A which is referred as suldow site I. Both the nanoparticle protrudes with aliphatic region at front end of the pocket at respective domain. This interaction helps to compare the relative binding grove residues at suldow site I and II respectively for each nanoparticle and has a large impact on only one side of HSA for respective nanoparticle that could correspond to drug binding site. Furthermore, in case of TiO_2_-HSA interaction extensive rearrangement of the H-bond network occurs between residues Arg 257, Ala 258, Ser 287, His 288, Leu 283, Ala 254, Tyr 150 with nanoparticle and this may result in increase in pocket volume for drug like azapropazone which are specific for suldow site I [13]. The success rate of docking for region predicted for the binding in case of both CuO np-HSA and TiO_2_ np-HSA interaction was considered by an observed binding energy as it was found to be ΔG<0.

The general anesthetic propofol has also been shown to bind to Sudlow’s site II (subdomain IIIA) in a cavity that able to host the diiodophenol ring of a thyroxine molecule as well [20]. The general anesthetic halothane also binds to Sudlow's site II. Since, we have also predicted that CuO nanoparticle also bind to Sudlow's site II i.e, subdomain III A thus can be used with thyroxine in exploring the pharmacokinetics, and may increase vulnerability of thyroxin in blood stream after binding with serum albumin protein, subsequently, can also serve as inhibitor for thyroxine to bind with albumin protein and to understand its mechanism more clearly. However, TiO_2_ np bind to site I there-by can be use as competitive inhibitor for phenylbutazone by competing at suldow site 1 and can protrude in drug binding efficacy. Moreover, indomethacin (IMN) azapropazone (AZP) drug can bind to subdomain II A. studies have shown that because of nanomaterials property large surface to volume ratio they are used frequently in drug delivery system. Considering TiO_2_ NPs binding mode with subdomain II A, TiO_2_ Nps can be used as nanocomposite for indomethacin (IMN) as well as with azapropazone (AZP) drug in a drug delivery system [12].

Subdomain III A in HSA has also been reported to possess esterase like activity (hydrolysis of p-nitrophenol esters) [21]. As discussed CuO nanoparticle bind to subdomain III A thus binding of these nanoparticles to this region may influence esterase like activity of Serum albumin protein. Thus, CuO np can be variably used with different alkaloids for its effectiveness by targeting esterase like activity of serum albumin protein [21].

The 4-Phenylbutyric acid (4-PBA) is a drug used in disease conditions like type 2 diabetes, obesity and neurodegeneration [22]. It alleviates endoplasmic reticulum stress by assisting protein folding. The molecular docking study on 4-PBA explores the binding nature of 4-PBA with human serum albumin (HSA) and reported that 4-PBA has high binding specificity to Suldow Site II (subdomain IIIA). The suldow site II also knows as Fatty acid binding site 3, (subdomain III A). We can say based on our analysis that CuO nanoparticles bind to subdomain III A i.e fatty acid binding site 3 thus CuO can also be study for drug delivery approach for 4-PBA in-order to understand its kinetics. Moreover, CuO could also be applied with application like micelles formation to overruled fatty acid binding potential with serum albumin protein by targeting fatty acid binding site 3 in HSA protein.

## 5 Conclusions

HSA comprises of three homologous domains (I, II, and III) that assemble to form a cordate molecule. The hydrophobic pockets at subdomains IIA and IIIA is the main regions of ligand interaction with IIIA having the highest affinity for the interaction. In our study the whole region of HSA was selected in order to predict binding of HSA with CuO and TiO_2_ nanoparticle. We have exploited CuO nanoparticle bind to site II, subdomain III A and TiO_2_ nanoparticle bind to site I located at subdomain II A of HSA protein. Thus, it can be inferred that both CuO and TiO_2_ nanoparticles showed HSA binding properties, with TiO_2_ nanoparticle having a slightly higher affinity towards HSA. This is attributed on the basis of the binding energy which is less in case of TiO_2_ nanoparticle as compared to CuO nanoparticle.
